# P16 OPAT service in a tertiary neurosurgery centre: a review of patient outcomes

**DOI:** 10.1093/jacamr/dlaf239.020

**Published:** 2026-01-05

**Authors:** Sian Lofthouse, Susan Larkin, Joanne Leahy, David Carter

**Affiliations:** The Walton Centre NHS Foundation Trust, Liverpool, UK; The Walton Centre NHS Foundation Trust, Liverpool, UK; The Walton Centre NHS Foundation Trust, Liverpool, UK; The Walton Centre NHS Foundation Trust, Liverpool, UK

## Abstract

**Background:**

The Walton Centre NHS Foundation Trust is a specialist hospital providing neurology and neurosurgery services to a large geographical area. The OPAT multidisciplinary team consists of a Consultant Neurosurgeon, Consultant Medical Microbiologist, Antimicrobial Pharmacist and Specialist Nurse. All patients prescribed either oral and parenteral antibiotics for complex neurosurgical infections are monitored through the OPAT clinic following discharge. Blood sampling is performed weekly or fortnightly and patients reviewed with a telephone or face to face appointment.

**Methods:**

All infection details and patient outcomes are maintained within a secure Excel database. Site and origin of infection, causative organism, treating antibiotic, route of antibiotic, adverse effects and overall outcomes were analysed.

**Results:**

A total of 57 patients used the OPAT service in 2024. Twenty-eight percent of patients were treated for cranial infections and 72% were treated for spinal infections. Most of the infections were post-operative compared to spontaneous infections. A total of 21 patients received IV antibiotics, and 36 patients were on oral therapy alone. Ceftriaxone was the most used IV antibiotic and co-trimoxazole and doxycycline were the most used oral antibiotics. Eighty-one percent of patients completed treatment as planned, 10% required a change in therapy due to adverse effects and 2% required a treatment extension. The adverse effects reported included GI side effects, deranged liver function tests, acute kidney injury and neutropenia. Three patients were readmitted: one for adverse effects and two for further surgery.

**Conclusions:**

Since the OPAT service was established, it continues to provide assurance of the safe and effective management of neurosurgical infections at home.

